# Green Synthesis of Gold Nanoparticles Using Upland Cress and Their Biochemical Characterization and Assessment

**DOI:** 10.3390/nano12010028

**Published:** 2021-12-23

**Authors:** Noah Hutchinson, Yuelin Wu, Yale Wang, Muskan Kanungo, Anna DeBruine, Emma Kroll, De’Jorra Gilmore, Zachary Eckrose, Stephanie Gaston, Phoebe Matel, Matey Kaltchev, Anne-Marie Nickel, Subha Kumpaty, Xiaolin Hua, Wujie Zhang

**Affiliations:** 1 Department of Biomedical Engineering, Milwaukee School of Engineering, Milwaukee, WI 53202, USA; Hutchinsonn@msoe.edu; 2 Department of Obstetrics, Shanghai First Maternity and Infant Hospital, Tongji University School of Medicine, Shanghai 201204, China; wuyuelin_wyl@163.com; 3 Department of Mechanical Engineering, University of Milwaukee, Milwaukee, WI 53211, USA; yalewang815@gmail.com; 4 Department of Physics and Chemistry, Milwaukee School of Engineering, Milwaukee, WI 53202, USA; kanungom@msoe.edu (M.K.); debruinea@msoe.edu (A.D.); krolle@msoe.edu (E.K.); gilmore@mose.edu (D.G.); zakeckrose@icloud.com (Z.E.); gastonsw@alumni.msoe.edu (S.G.); matelpr@alumni.msoe.edu (P.M.); kaltchev@msoe.edu (M.K.); nickel@msoe.edu (A.-M.N.); 5 Biomolecular Engineering Program, Milwaukee School of Engineering, Milwaukee, WI 53202, USA; 6 Department of Mechanical Engineering, Milwaukee School of Engineering, Milwaukee, WI 53202, USA; kumpaty@msoe.edu

**Keywords:** green synthesis, nanoparticles, catalysis, cytotoxicity, upland cress

## Abstract

This research focuses on the plant-mediated green synthesis process to produce gold nanoparticles (Au NPs) using upland cress (Barbarea verna), as various biomolecules within the upland cress act as both reducing and capping agents. The synthesized gold nanoparticles were thoroughly characterized using UV-vis spectroscopy, surface charge (zeta potential) analysis, scanning electron microscopy-energy-dispersive X-ray spectroscopy (SEM-EDX), atomic force microscopy (AFM), attenuated total reflection Fourier transform infrared spectroscopy (ATR-FTIR), and X-ray diffraction (XRD). The results indicated the synthesized Au NPs are spherical and well-dispersed with an average diameter ~11 nm and a characteristic absorbance peak at ~529 nm. EDX results showed an 11.13% gold content. Colloidal Au NP stability was confirmed with a zeta potential (ζ) value of −36.8 mV. X-ray diffraction analysis verified the production of crystalline face-centered cubic gold. Moreover, the antimicrobial activity of the Au NPs was evaluated using Gram-negative *Escherichia* *coli* and Gram-positive *Bacillus megaterium*. Results demonstrated concentration-dependent antimicrobial properties. Lastly, applications of the Au NPs in catalysis and biomedicine were evaluated. The catalytic activity of Au NPs was demonstrated through the conversion of 4-nitrophenol to 4-aminophenol which followed first-order kinetics. Cellular uptake and cytotoxicity were evaluated using both BMSCs (stem) and HeLa (cancer) cells and the results were cell type dependent. The synthesized Au NPs show great potential for various applications such as catalysis, pharmaceutics, and biomedicine.

## 1. Introduction

Gold nanoparticles have potential applications in numerous fields including chemical/biochemical industries, textiles, energy, bioremediation, bioimaging, biosensorics, controlled delivery of therapeutic agents, and agriculture due to their unique properties [1,2]. In particular, the characteristic chemical, biological, optical, and electrical properties of gold nanoparticles have been utilized for nano-catalysis and nanotheranostics, to provide novel routes for chemical reactions, and to offer a personalized approach for medical diagnosis and treatment [3,4,5,6,7]. Specifically, biocompatible gold nanoparticles exhibit unique surface interactions and can be functionalized through the attachment of numerous components (drugs, targeting moieties, radioisotopes, DNA, fluorescent dyes, linkers, polyethylene glycol, and others) to offer multimodal approaches to modern biomedical processes [8,9].

Several traditional methods for the production of gold nanoparticles, including chemical and physiochemical methods, have been utilized for these purposes. However, many of these pathways draw concern due to their adverse environmental impact, high cost and energy consumption, as well as the potentially limited applications of the produced nanoparticles. Alternatively, biological and green synthesis methods utilize microorganisms as “biomachinery” or naturally occurring biomolecules from plant extract that serve as reducing and capping agents in a bottom-up synthesis approach [10]. This is advantageous over traditional methods as it provides versatile, biocompatible nanomaterials through an environmentally-conscious and cost-effective approach. To date, various organisms including plants, bacteria, algae, and fungi have been found to contain the phytochemicals necessary for the green synthesis and stabilization of nanoparticles. Extracts used for green synthesis of Au NPs include *Coffea arabica* (coffee), *Solanum nigrum* (black nightshade), *Nasturtium officinale* (watercress), *Brazilian red propolis* (honeybee product), *Litsea cubeba* (May Chang), *Chlorella vulgaris* (algae), *Mimosa tenuiflora* (Jurema), and *Ziziphus zizyphus* (Jujube) [6,11,12,13,14,15].

Upland cress (*Barbarea verna*) is a widely available biennial leafy green in the *Brassicaceae* family which has been found to contain a high content of synthetically viable phytochemicals including ascorbic acid, carotenoids, and tocopherols that are potentially useful for the reduction and stabilization of gold nanoparticles [16]. Phenolics such as flavonols mainly contribute to the reducing process [17]. Phenolics and other plant metabolites (such as sugars and enzymes) are responsible for stabilization and capping [18]. Recently, upland cress has been used to successfully synthesize silver nanoparticles [19]. In this work, a novel green synthesis process to produce gold nanoparticles (Au NPs) using upland cress, for different applications from silver nanoparticles, was developed and optimized. It was hypothesized that the nanoparticles formed using this method would show favorable stability due to the presence of phytochemical based capping agents present in the upland cress extract. Furthermore, the synthesized Au NPs would show antimicrobial properties and biocompatibility, as well as serve as a catalyst. This novel green synthesis method offers a cost-effective and environmentally friendly counterpart to traditional methods for biocompatible nanoparticle synthesis.

## 2. Materials and Methods

### 2.1. Materials

Upland cress (B&W Quality Growers, Fellsmere, FL, USA) was purchased from Whole Foods, a local grocery store, and stored at 4 °C. Gold (III) chloride trihydrate (HAuCl_4_; 520918), 4-nitrophenol (C_6_H_5_NO_3_; 241326), Folin-Ciocalteu reagent (F9252), gallic acid (G7384), and sodium borohydride (NaBH_4_; 80637300) were obtained from Millipore Sigma (St. Louis, MO, USA) and were used as received.

### 2.2. Analysis of the Upland Cress Extract

The total phenolic content of the sample was determined by the Folin-Ciocalteu method [20]. A 20 µL aliquot of upland cress extract was added to 1.58 mL water and 100 µL of the Folin-Ciocalteu reagent. After 5 min, 300 µL of 20% sodium carbonate solution was added to the mixture and agitated for 10 min. After sitting in the dark for 2 h at 22 °C, the absorbance was measured at 765 nm using UV spectrophotometer (Genesys^TM^ 150; Thermo Fisher Scientific, Waltham, MA, USA). Different concentrations of gallic acid were used to prepare the calibration curve (R^2^ = 0.9915). The total phenolic content was expressed as µg gallic acid equivalent (GAE) per mL extract.

The concentration of ascorbic acid (vitamin C) in the upland cress was analyzed through iodometric titration [21]. Briefly, 4 mL of upland cress extract, 0.2 mL 0.5% starch, 1 mL 0.6 M potassium iodide, 1 mL of 1 M HCl, and 30 mL of DI water were mixed in a flask. The mixture was titrated using 0.002 M potassium iodate with the first permanent trace of blue-black color as an indicator of the endpoint.

### 2.3. Green Synthesis of Au NPs

**Scheme 1:**
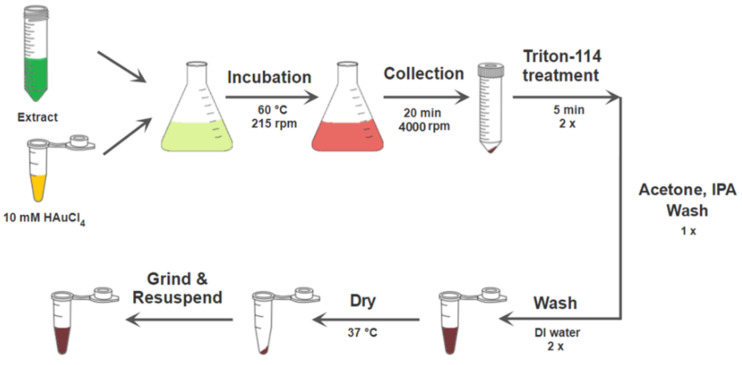
Process for the green synthesis of Au NPs using upland cress.

The green synthesis and purification protocols(Scheme 1) were based on previous work [19] with modifications, in the extraction process (blending instead of boiling was used). Briefly, 10 g of upland cress were blended in 100 mL deionized (DI) water for 15 s. The mixture was then vacuum filtered twice using Whatman filter paper and centrifuged at 4000 rpm for 5 min to produce the extract (supernatant). Extract (5 mL), 10 mM gold (III) chloride trihydrate (0.4 mL), and DI water (35 mL) were mixed and then placed in a shaker (215 rpm) at 37 °C.

For purification, sonication and two more solvents were utilized to achieve a more thorough process in comparison to previous work [19]. The nanoparticles were collected through centrifugation (4000 rpm, 20 min). The particles were suspended in DI water (10 mL) and sonicated for 5 min. The suspension was then centrifuged (4000 rpm, 20 min). Subsequently, the collected nanoparticles were subjected to a series of wash/vortex and centrifugation cycles using Triton X-114 (0.75 µL/mL DI water), acetone, isopropyl alcohol, and DI water respectively. Finally, the nanoparticles were oven dried at 45 °C, ground using a mortar and pestle, and stored at −20 °C for future use.

### 2.4. Characterization of Au NPs

UV-Vis spectroscopy (Genesys^TM^ 150; Thermo Fisher Scientific, Waltham, MA, USA) was used to study the effects of incubation time on the green synthesis process. The peak absorbance wavelength was determined using the scanning mode (450–650 nm). Zeta (ζ) potential of the Au NPs (0.4 mM) was measured using a Zetasizer Nano ZS (Malvern, Westborough, PA, USA). Scanning electron microscopy (FE-SEM, Hitachi S-4800 ultra-high resolution cold cathode field emission scanning electron microscope, Kefeld, Germany) was used to image Au NPs (40 mM) that were dried and mounted to aluminum stubs. At the same time, energy dispersive X-ray spectroscopy (EDX, Noran (Si(Li))detector, Thermo Fisher Scientific, Waltham, MA, USA) was used to verify the elemental composition of the Au NPs. Atomic force microscopy (AFM) images, using 1.25 mM Au NP solution, were taken using contact mode on a Bruker MultiMode atomic force microscope (Billerica, MA, USA) with a Veeco Nanoscope IIIa controller (Santa Barbara, CA, USA). Au NPs (oven-dried at 45 °C) were also analyzed using attenuated total reflection Fourier transform infrared (ATR-FTIR; MIRacle 10, IR-Tracer 100; Shimadzu, Kyoto, Japan) spectroscopy. Lastly, powder X-ray diffraction analysis was performed using a Bruker D8 Discover X-ray diffractometer (Billerica, MA, USA) to confirm the crystalline structure of the Au NPs.

### 2.5. Catalysis of the Reduction of 4-Nitrophenol

Sodium borohydride (200 mM; over ice) and 4-nitrophenol (2.0 mM) solutions were freshly prepared. Then, 50 µL 4-nitrophenol, 5 µL Au NPs (80 mM), and 2 mL DI water were gently mixed in a cuvette. After this, 25 µL NaBH_4_ was added into the cuvette immediately before starting measurements. Scans were performed every minute for 30 min using a UV-Vis spectrophotometer (250–550 nm) [22].

### 2.6. Antibacterial Activity Testing

The antibacterial effects of Au NPs on both Gram-negative *Escherichia coli* (Item #: 470176-528, Ward’s Science, Rochester, NY, USA) and Gram-positive *Bacillus megaterium* (Item #: 15-4900, Carolina Biological Supply Company, Burlington, NC, USA) bacteria were evaluated using the agar disc diffusion method [23,24]. *E. coli* and *B. megaterium* were cultured in nutrient broth media (37 °C, 24 h) and inoculated onto agar plates (Mueller-Hinton growth medium). Diffusion discs were dipped into varying concentrations of Au NP solution (0.50, 0.25, 0.10, 0.05 mM) and placed on the inoculated plates. Ampicillin discs (10 mcg, AMP10-1815; Carolina Biological Supply Company, Burlington, NC, USA) and blank discs were added to the plates and served as positive and negative controls, respectively. The plates were incubated (37 °C, 24 h) and the antibacterial inhibition zones were analyzed.

### 2.7. Cytotoxicity and Cellular Uptake Studies

Bone marrow mesenchymal stem cells (BMSCs; MUBMX-01001, Cyagen, Santa Clara, CA, USA) and HeLa cells (Shanghai Key Laboratory of Maternal Fetal Medicine, Shanghai, China) were cultured in Dulbecco’s Modified Eagle’s Medium (DMEM)/F-12 (Gibco, Grand Island, NY, USA) and DMEM, separately. The medium was supplemented with 10% fetal bovine serum, 100 U ml^−1^ penicillin, and 100 mg/L streptomycin (Gibco, Grand Island, NY, USA). The cells were cultured in a humidified CO_2_ incubator (5%) at 37 °C. For the cytotoxicity study, cells were seeded in 96-well plates at a density of 10,000 cells/well. After 24 h incubation, culture medium was replaced using fresh medium containing various concentrations of Au NPs (0.10, 0.25, 0.50, 1.00, 1.50, 2.00, 2.50 mM). The cells were then rinsed twice with PBS after 24 h and medium containing CCK8 (10 µL/100 µL medium; Beyotime Institute of Biotechnology, Shanghai, China) was added. After 2 h, 100 µL medium of each well was transferred to a new 96-well plate and the absorbance was determined at 450 nm using a micro plate reader (Bio-Rad 680, Bio-Rad; Hercules, CA, USA). Cell viability was determined using the absorbance ratio of an experiment well to the average of the control wells (i.e., cell culture medium only).

To assess cellular uptake, at 70–80% confluency, cells were cultured in fresh medium containing 1 mM Au NPs for 24 h. Then, cells were detached using 0.25% trypsin-ethylenediaminetetraacetic acid (EDTA) (Gibco, Grand Island, NY, USA) digestion, rinsed twice in cold PBS, and collected through centrifugation. Cells were then fixed using 2.5% glutaraldehyde for 2 h. Subsequently, the cells were washed and fixed with cacodylate buffer and osmium tetroxide (2%), respectively, dehydrated with 70–100% acetone and embedded and cut in a film (70 nm) using an ultra-microtome. After a uranyl acetate-lead citrate double staining, the samples were observed under TEM (H-600, Hitachi; Tokyo, Japan).

## 3. Results and Discussion

### 3.1. Total Phenolic and Ascorbic Acid Content

When plants contain higher total phenolic content, they possess stronger antioxidant activity [25]. Ascorbic acid also contributes to high levels of antioxidant capacity of upland cress [19]. Hence, it is critical to determine the total phenolic and ascorbic acid content. The total phenolic and ascorbic acid concentrations were determined to be 163.3 ± 1.5 µg GAE/mL extract and 24.5 ± 1.1 µg/mL extract, respectively. The high phenolic and ascorbic acid content potentially contributed to reduction and capping during nanoparticle synthesis. 

### 3.2. Effects of Incubation Time on the Green Synthesis Process

**Figure 1:**
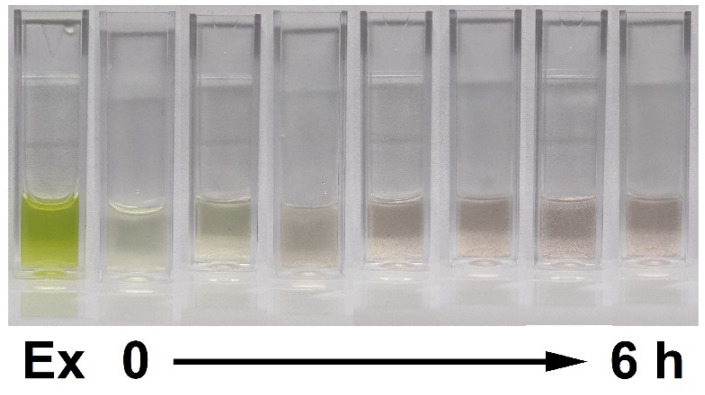
Solution color change of the upland cress extract and gold (III) chloride trihydrate mixture during the incubation period. Time interval: 1 h.

**Figure 2:**
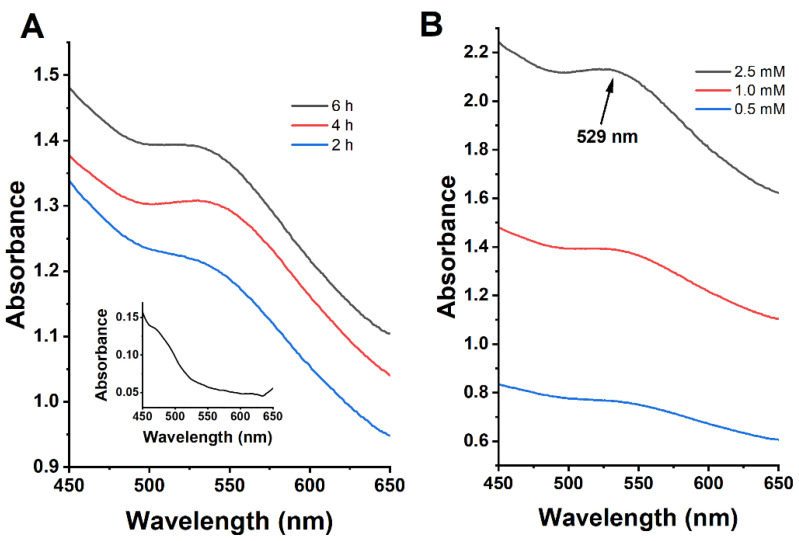
UV-Vis spectra of the synthesized Au NPs. (**A**) different incubation times during the green synthesis process (1.0 mM); insert: 0 h incubation (i.e., mixture of extract and HAuCl_4_ in DI water). (**B**) different concentrations of synthesized Au NPs (6-h incubation).

To improve the extraction efficiency and consistency of the synthesis process, the previous procedure [19] was modified. Specifically, blending was used to prepare the ex-tract rather than boiling, as boiling showed inconsistencies. The unique optical properties of Au NPs are primary indicators for confirming the successful synthesis of nanoparticles. The apparent color transition from pale green to wine red color (Figure 1) is indicative of the formation of gold nanoparticles due to the surface plasmon resonance phenomenon [26]. As the nanoparticles grow, the absorption wavelengths become longer and redder. The color and intensity changes reflect the formation and growth of Au NPs, respectively [26]. There was no significant color change observed after 4 h. To further investigate the effects of incubation time on the green synthesis process, 2-, 4-, and 6-h-long periods were selected based on a previous study [19]. As shown in Figure 2A, a characteristic absorption peak is visible for each nanoparticle sample prepared with different incubation times. A right shift and intensity increase of the characteristic absorbance peak between the 2- and 4-h samples indicate the growth of Au NPs and potential formation of agglomerates. After 4 h, there was no significant peak shift. Comparing with the control (0 h incubation, insert of Figure 2A), the absorbance remains in the low (450–650 nm) wavelength range. No absorbance peak is visible ~530 nm. After 4 h, there was no significant peak shift. Based on the results, a 6-h incubation time was chosen for the nanoparticle synthesis. UV-Vis spectroscopy results (Figure 2B) indicated a characteristic absorption peak of about 529 nm, which is within the characteristic range for gold nanoparticles (~500–550 nm). The concentration-dependence of particle formation was also observed when various concentrations of Au NPs (0.5, 1.0, 2.5 mM) were used (Figure 2B). The observed time-dependence and concentration-dependence of Au NP formation is consistent with literature [27,28].

### 3.3. Morphology, Chemical Composition, and Surface Charge

**Figure 3:**
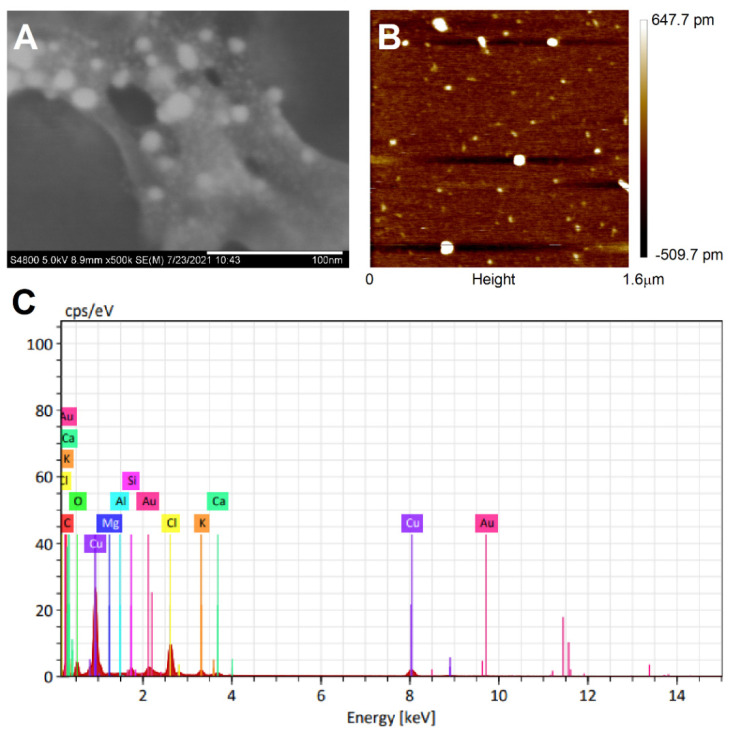
SEM (**A**) and AFM (**B**) images and EDX spectrum (**C**) of the synthesized Au NPs. SEM image scale bar: 100 nm.

Both SEM and AFM were used to examine the morphology and size/size distribution of the Au NPs. SEM and AFM offer straightforward visualization of metallic nanoparticles due to their high resolution. Au NPs are indicated by the AFM and SEM as the bright spots in either image. SEM imaging (Figure 3A) revealed the production of spherical Au NPs with uniform size (10.7 ± 2.2 nm) and without aggregation. AFM imaging (Figure 3B) further confirmed the production of well dispersed spherical nanoparticles with a narrow size distribution. EDX results (Figure 3C) indicate an 11.13% gold composition by mass. Carbon and oxygen peaks within the spectrum indicate the presence of phytoconstituents, organic capping agents associated with the upland cress extract. Other inorganic elemental species such as calcium, potassium, chlorine, and magnesium were observed and their presence can be attributed to their high content in upland cress [12,29]. The presence of copper in the EDX spectrum was observed due to the conductive adhesive used for SEM imaging. Zeta potential measurements were performed to assess the surface charge and stability of the synthesized Au NPs. Zetasizer readings provided an average zeta (ζ) potential of −36.8 mV, implying favorable colloidal stability [30]. The apparent stability is most likely due to the phytochemical capping of the Au NPs.

**Figure 4:**
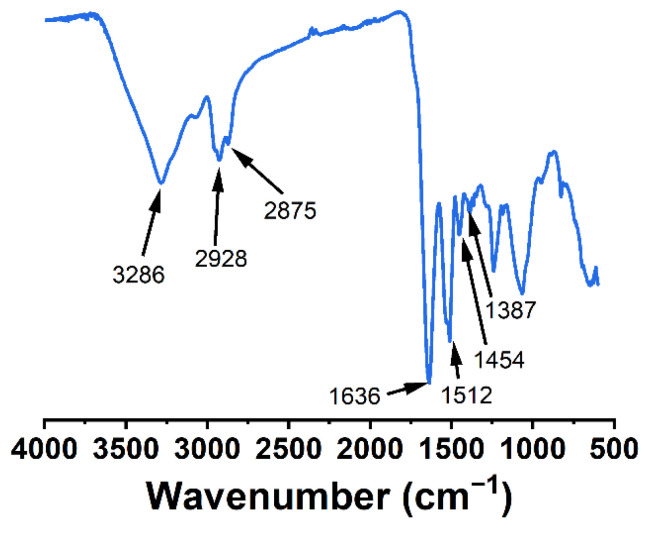
ATR-FTIR spectrum of the synthesized Au NPs.

The ATR-FTIR spectrum of Au NPs is shown in Figure 4. Functional groups were assigned to the corresponding spectral bands based on literature [15,31]. The bands include 3286 cm^−1^ (–OH stretching of phenolics and other phytochemicals); 2928 cm^−1^ and 2875 cm^−1^ (–CH stretching of alkanes); 1636 cm^−1^ (including –NH bending and –C=O); 1512 cm^−1^ (–CH of alkanes and –NO of nitro-compounds); 1454 cm^−1^ (including –OH bending –C=O of phenolics and other phytochemicals); and 1387 cm^−1^ (–CN stretching of aromatic amine group). Based on the results, the presence of stabilizing/capping agents (phenolics and other phytochemicals of upland cress extract) on the Au NPs was confirmed [15].

### 3.4. Crystal Structure of Au NPs

**Figure 5:**
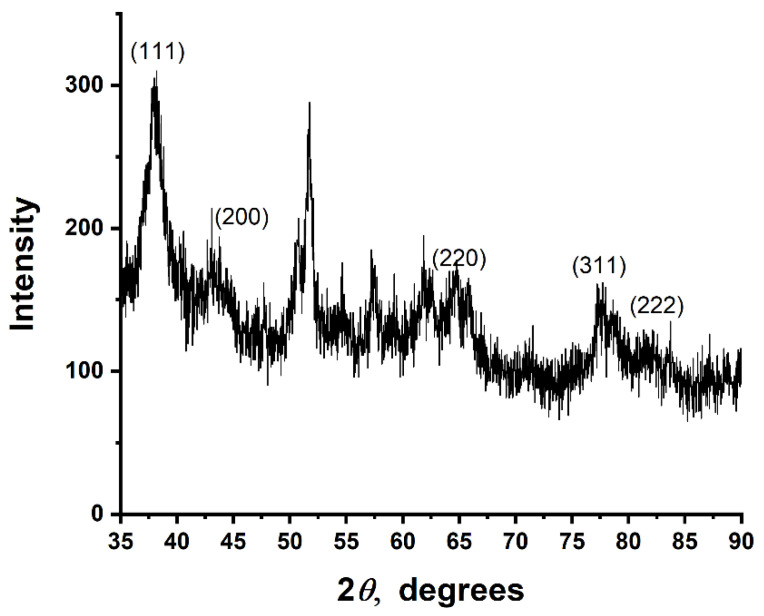
XRD spectrum of the synthesized Au NPs.

Powder XRD analysis (Figure 5) provided diffraction peaks at 2*θ* angles 38.20°, 43.73°, 64.77°, 77.72°, and 82.09° corresponding to the crystalline gold atomic planes (111), (200), (220), (311), and (222) confirming the expected face-centered cubic structure (JCPDS Card No. 96-901-1613). Unassigned diffraction peaks are presumed to be related to the production of bio-organic crystallite phases on the surface of the Au NPs [32,33]. Peak broadening observable in the XRD pattern can be attributed to the scale of the measured crystallites as explained by the Scherrer equation (Equation (1)) [19,34].

(1) $$D=\frac{k\lambda }{\beta \;\cos\left(\theta \right)}$$

where k = 1, λ = 0.1542, β is the full width at half maximum, and θ is the diffraction angle. Using 2θ values of 38.20° and 64.77°, the nanoparticle size was determined to be approximately 13 nm, which is similar to the SEM and AFM results.

### 3.5. Catalysis of the Reduction of 4-Nitrophenol

**Figure 6:**
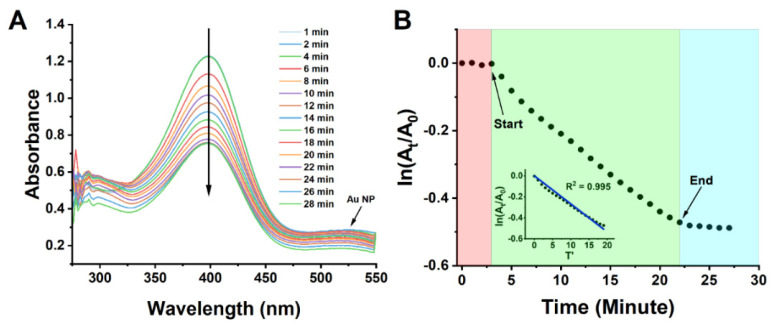
UV-vis spectra (**A**) at different time points and reaction kinetics. (**B**) The Au NP catalyzed reduction of 4-nitrophenol. T’ = T − 4.

The catalysis of the reduction of 4-nitrophenol is a commonly used reaction when testing the catalytic ability of nanoparticles [12,35,36,37]. Verification of nanoparticle catalysis is centered on the analysis of the extinction of the absorbance peak of 4-nitrophenol (400 nm) which indicates the catalyzed reduction of 4-nitrophenol to 4-aminophenol. Results show that over a 30-min measurement interval, the characteristic peak of 4-nitrophenol decreased substantially, verifying successful catalytic ability (Figure 6A). The catalysis was also verified through the reaction system color change from bright yellow to pale pink (color of Au NPs). The kinetics of the catalyzed reaction were analyzed according to the Langmuir-Hinshelwood mechanism for bimolecular surface reactions [6,38]. According to this general model, the reduction reaction occurs on the surface of the Au NPs. Here, borohydride ions (BH_4_^−^) adsorb to the surface and hydrogen species are formed via electron transfer. At the same time, 4-nitrophenol adsorbs to the surface. The 4-nitrophenol is then reduced to 4-aminophenol on the surface before detachment from the catalyst site [12]. The pseudo-first order equation used to analyze this process is shown as Equation (2).

(2) $$ln^{\left(\frac{A_{t}}{A_{0}}\right)}=-k_{app}t$$

Application of this relationship reveals an initial period of no reaction (4 min), followed by first-order reaction kinetics (linear region). The rate constant was derived from the linear region and was found to be 0.0267 min^−1^ (Figure 6B) with an R^2^ of 0.995. The initial period of no reaction is most likely attributed to the blockage of potential catalysis sites by capping molecules. This size of Au NPs is preferred for catalysis according to reference [39,40]. 

### 3.6. Antibacterial Activity

**Figure 7:**
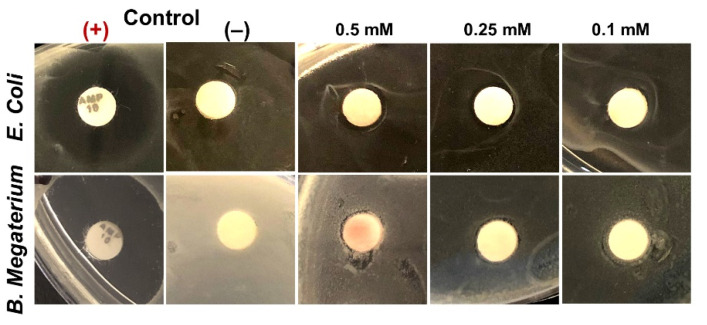
Antimicrobial testing results: top: *E. coli* and bottom: *B. megaterium*. The ampicillin and blank discs were used as positive and negative control, separately.

The analysis of inhibitory zones shows a dose-dependent and species dependent antibacterial effect on Gram-negative *E. coli* and Gram-positive *B. megaterium* (Figure 7*)*. Measurements of bacterial growth inhibition indicate the largest zones of inhibition occurring at Au NP doses of 0.25 mM (9 mm) and 0.5 mM (7.25 mm) for *E. coli* and *B. megaterium,* respectively. Analysis of the average inhibition zones across all trials indicate a higher antibacterial activity in the *B. megaterium* with an average of 7.3 mm versus an average of 6.6 mm seen with *E. coli.* The Au NPs appear to have lower antibacterial activity than Ag NPs using the same synthesis method [19]. It is reported that nanoparticles have been demonstrated to show a size and surface ligand dependent cytotoxic effect [24,41]. The small size of the synthesized Au NPs and the presence of bioorganic surface ligands related to the upland cress extract may contribute to the observed antibacterial activity. This antibacterial activity of Au NPs may be attributed to one or multiple mechanisms of Au NPs including the direct disruption of major internal cell function (ATP production, DNA replication, enzyme inhibition), the formation of toxic reactive oxygen species (ROS), as well as direct damage to the cellular membrane [41,42]. The antibacterial activity of Au NPs show promise for their biomedical applications with added infection prevention benefits.

### 3.7. Cytotoxicity and Cellular Uptake

**Figure 8:**
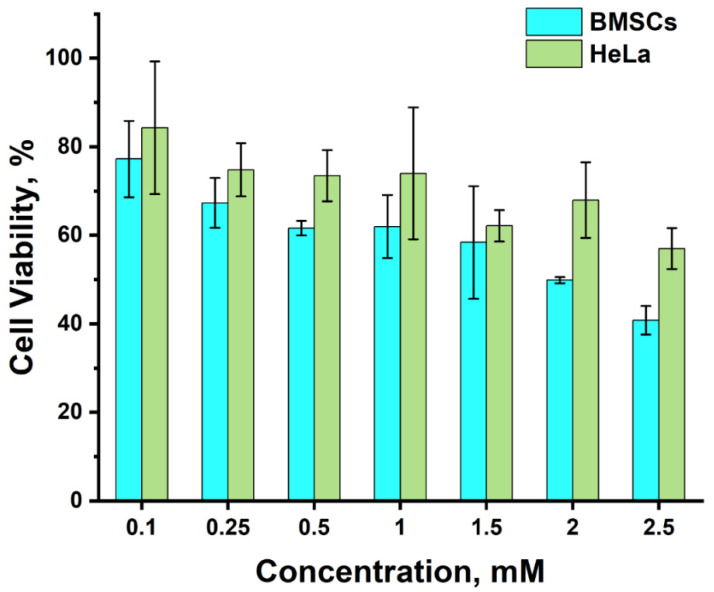
Cytotoxicity of Au NPs using both BMSCs and HeLa cells.

**Figure 9:**
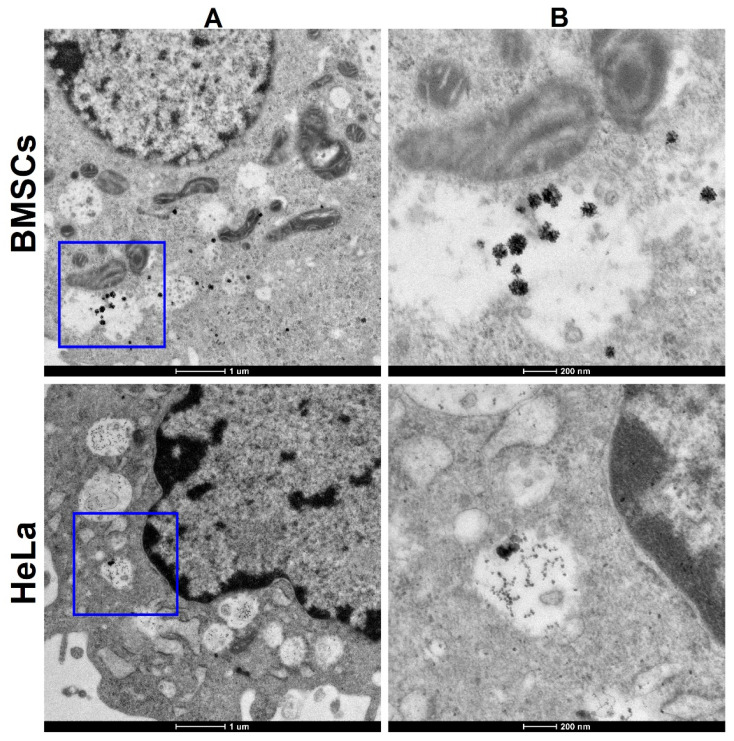
TEM images indicating internalization of Au NPs within BMSCs and HeLa cells. Enlarged images of the highlighted regions in column A are shown in column B. Scale bars: 1 µm (**A**) and 200 nm (**B**).

Considering the potential biomedical applications, both stem (BMSCs) and cancer (HeLa) cells were used to study the cellular uptake and cytotoxicity of Au NPs (Figure 8). BMSCs were studied for potential stem-cell based medicines, while HeLa cells were examined for prospective cancer treatment applications. A broad range (0.1–2.5 mM) of nanoparticle concentrations were tested and LC_50_ (50% lethal concentration—the concentration that kills 50% of the cells) were determined. The results showed that BMSCs (LC_50_ = 2 mM) are more sensitive to the Au NPs than HeLa cells (LC_50_ > 2.5 mM). The cell viability of the BMSCs dropped lower than 70% when the Au NP concentration reaches 0.1 mM while the HeLa viability remains higher than 70% until the concentration surpasses 1.0 mM. A recent study showed about 80% cell viability of PC3 cancer cells using Au NPs of similar size, which is close to our results (84%) [43]. A cell viability of ~79% for BMSCs was reported with spherical Au NPs (18 nm; 0.09 mM) [44] which is comparable to our results as well (77%; 0.1 mM). A recent study indicated that 15 nm Au NPs could affect the characteristic MSC marker expression (e.g., CD 105) and cell differentiation, especially when the concentration is higher than 9 µg/mL [45]. Cell damage and apoptosis may be explained by the generation of reactive oxidative stresses (ROS) [46]. Based on the cytotoxicity results a concentration of 1.0 mM was used for cellular uptake studies. TEM images of the cellular studies (Figure 9) indicate that nanoparticles are visible within the cells. The nanoparticles are mostly within membrane-bound vesicles such as endosomes (formed due to endocytosis). These results are consistent with previous publications which state that endocytosis (most likely receptor-mediated) is the primary route for cellular uptake [43,47]. For potential applications, the size of the Au NPs can be fine-tuned to adjust toxicity and cellular uptake, as it has been well studied that the size greatly affects the nanoparticle-cell interactions (e.g., cellular uptake efficiency and mechanism) (size-dependent cellular uptake and localization profiles of silver nanoparticles; size- and cell type-dependent cellular uptake, cytotoxicity and in vivo distribution of gold nanoparticles).

## 4. Conclusions

The study focused on the novel use of upland cress as a green synthesis agent for the production of gold nanoparticles. A blending method was used to extract the necessary phytochemicals (e.g., phenolics and ascorbic acid) within the upland cress to serve as a reducing agent for the reduction of gold (III) chloride trihydrate and a capping agent. The resultant purified particles were characterized using UV-Visible spectroscopy, SEM-EDX, AFM, Zetasizer, ATR-FTIR, and XRD. The results indicated the successful synthesis of Au NPs which were found to be spherical, well dispersed with an average diameter ~11 nm, and a characteristic absorbance peak at ~529 nm. A negative ζ-value of −36.8 mV indicated stability of the Au NPs while XRD analysis verified the production of crystalline face-centered cubic gold. Furthermore, the antimicrobial testing results demonstrated concentration-dependent antimicrobial properties of the Au NPs. The catalytic ability of Au NPs was demonstrated through the conversion of 4-nitrophenol to 4-aminophenol (first-order kinetics). Cellular uptake and cytotoxicity studies using both BMSCs and HeLa cells indicated the uptake of Au NPs into cells and cell type dependent cytotoxicity. This green synthesis method provides a simple, cost effective, green solution to produce gold nanoparticles and provides a promising source of functionalized nanoparticles for use in various applications.

## Data Availability

The data that support the findings of this study are available from the corresponding authors upon reasonable request.
